# Actinomycosis Presenting as an Abdominal Mass in a Child

**Published:** 2011-03-10

**Authors:** Rahsan Özcan, Emil Mammadov, Emrah Aydin, Ibrahim Adaletli, Tugçe Esen, Sergülen Dervisoglu, Sinan Celayir

**Affiliations:** Istanbul University, Cerrahpasa Medical Faculty, Departments of Pediatric Surgery Istanbul, Turkey; Istanbul University, Cerrahpasa Medical Faculty, Departments of Pediatric Surgery Istanbul, Turkey; Istanbul University, Cerrahpasa Medical Faculty, Departments of Pediatric Surgery Istanbul, Turkey; Istanbul University, Cerrahpasa Medical Faculty, Departments of Pediatric Surgery, Istanbul, Turkey; 1 Istanbul University, Cerrahpasa Medical Faculty, Departments of Radiology Istanbul, Turkey; 2 Istanbul University, Cerrahpasa Medical Faculty, Departments of Pathology Istanbul, Turkey

**Keywords:** Abdominal actinomycosis, Abdominal trauma, Mass abdomen, Actinomycosis

## Abstract

Abdominal actinomycosis in childhood period is very rare and a relation to trauma is not well established. Herein we report a case that appeared subsequent to abdominal trauma. A 17 years old boy presented with left lower quadrant abdominal mass and signs of acute abdomen. The symptoms of abdominal discomfort began after a fall from height 3 months before admission. There were signs of acute abdomen at physical examination. Ultrasound of abdomen demonstrated a mass; CT scan findings pointed to a suspicious “internal hernia”. An emergency laparotomy was performed. During surgery, a mass located over sigmoid colon and infiltrating the lateral abdominal wall was found. It was removed en bloc with the adjacent omentum. Except for the thickened sigmoid colon, no other pathologies were present at laparotomy. The pathology specimen revealed the actinomyces infection. The patient was treated with oral penicillin after discharge and the follow-up was uneventful. We advocate, keeping the actinomyces infection in mind in cases presenting with abdominal mass of unknown origin in childhood period.

## INTRODUCTION

Abdominal actinomycosis is a rare condition, usually misdiagnosed as abdominal mass imitating a malignant tumor. Management usually consists of surgery followed by medical treatment with high dose penicillin [1, 2, 3, 4, 5, 6]. The diagnosis is confirmed with histopathological examination. The childhood presentation is also very rare. Herein we report a patient who presented with abdominal mass after trauma.

## CASE REPORT

A 17-year-old boy had a history of fall from fourth floor of a building three months back. There was no history of unconsciousness. The clinical examination, laboratory investigations and CT scan were reported as unremarkable at that time. The patient was discharged from hospital after 12 hours of observation.

After a month, he experienced abdominal pain and symptoms gradually progressed. The acute exacerbation resulted in patient reporting to the emergency room of our hospital. On physical examination, there was extensive abdominal tenderness and muscle guarding with a painful mass in the left lower quadrant. The body temperature was normal. Leukocyte count was 16400. The plain abdominal film was reported as normal. Ultrasound (US) demonstrated the mass which was also confirmed on CT scan. Radiologist interpreted the condition as an internal herniation of the intestines (Fig. 1).

**Figure:**
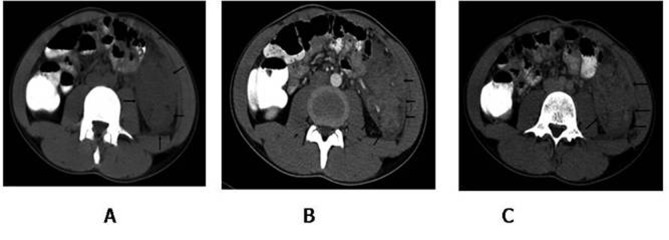
Figure 1: Axial computed tomography image show a soft tissue mass, located between left psoas muscle and lateral abdominal wall. The lesion partially surrounds the sigmoid colon (A). Contrast enhanced CT arterial (B) and venous (C) phase show marked heterogeneous involvement.

As the signs of acute abdomen persisted, emergency laparotomy was performed after adequate fluid replacement and administration of broad-spectrum antibiotics. At exploration, a mass located over sigmoid colon and infiltrating the lateral abdominal wall with a small abscess formation was found. Abscess drained spontaneously during manipulation. The mass was removed completely with the adjacent omentum. No other pathologies were found at except for the thickened sigmoid colon (Fig. 2). The histopathological examination revealed actinomyces infection (Fig. 3a, 3b). Microbiological examination did not show growth of this organism. Postoperative period was uneventful and the patient discharged on oral penicillin

**Figure:**
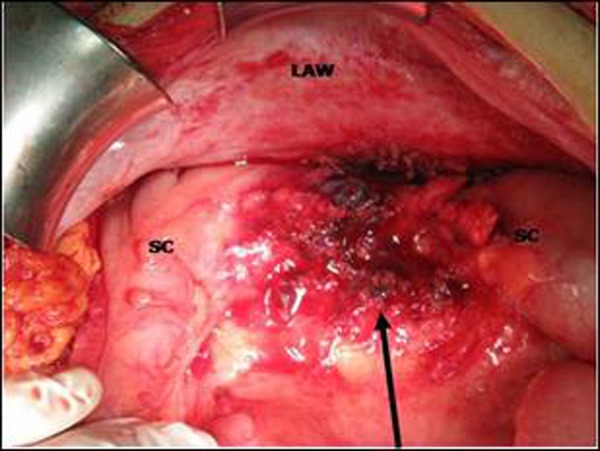
Figure 2: Arrow indicates the location of removed mass. It was over sigmoid colon (SC) and lateral abdominal wall (LAW)

**Figure:**
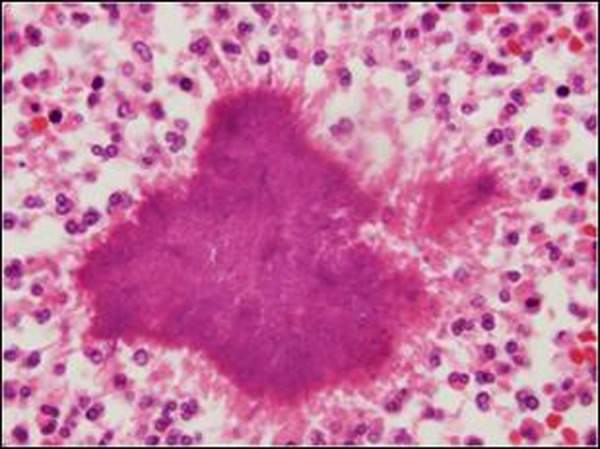
Figure 3a: A colony of actinomyces and mixed inflammatory response at the periphery which include histiyocytes, lymphocytes, plasmocytes, eosinophils and neutrophils.

**Figure:**
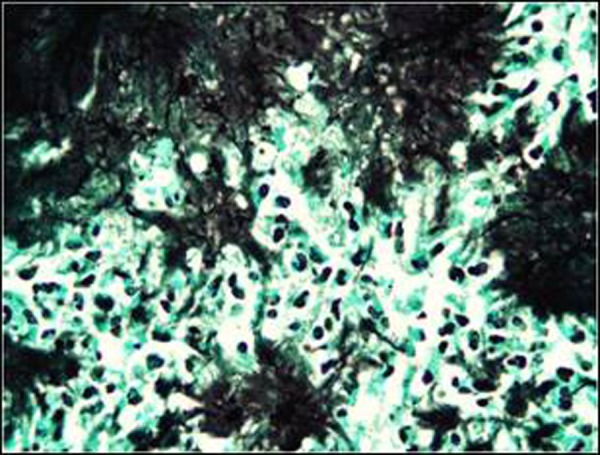
Figure 3b: Colony of filamentous microorganisms stain black with Grocott’s methenamin silver conventional staining method ( magnification x100 immertion )

## DISCUSSION

 Abdominal actinomycosis is a rare condition caused by anaerobic gram-positive bacteria habituating in human oral flora, usually diagnosed as abdominal mass. Three major locations for actinomycosis are described: cervicofacial, thoracic and abdominal. Abdominal involvement occurs in only 20% of cases. This pathology is mostly seen in women using intrauterine devices and is rarely reported in childhood. It can also mimic appendicitis, malignant diseases, tuberculosis and inflammatory bowel disease [1, 6, 7, 8, 9, 10].

Although medical treatment is suggested by some authors, most of the patients require surgery at the primary presentation. The diagnosis is mainly confirmed with histopathological examination.

Medical treatment with high dose injectable penicillin followed by oral penicillin is recommended for several weeks after discharge. Ultrasound and CT examinations can detect the mass, but this mass can be easily misinterpreted as in our case. Although experienced radiologist can suspect the pathology, still the preoperative diagnosis is difficult [4].

Rarity of the abdominal actinomycetes infection during childhood period overrules making an adequate algorithm for the treatment in this period. Therefore, the management is usually similar to adult cases. The main aim at operation should be to avoid unnecessary extensive resections, and restriction to the removal of the mass alone. This could be achieved with the help of frozen section during surgery and preoperative suspicion of the entity though very rare [6]. In our case, we did not have frozen section; but the atypical location without any obstruction, and absence of any gross infiltration to the corresponding tissues especially to the sigmoid colon, made us to decide to the removal of the mass alone.

A relation to trauma is rarely reported and the mechanism is not clearly understood. Our patient had a history of fall before hospitalization and the symptoms were on the left lower quadrant. Therefore, it seems that the mass formation was most likely related to this fall event.

Optimal therapy includes wide excision of necrotic, infected tissue and debris followed by an intense protracted antibiotic therapy. Intravenous 10-20 million units’ daily aqueous penicillin followed by 2-15 million units/day orally for a minimum of 2 months and long follow-up is recommended [4]. Triple antibiotics were administered in our case before confirmation of diagnosis. The therapy continued with oral penicillin.

In conclusion, abdominal actinomycosis is difficult to diagnose preoperatively and surgery is required in most of the cases. CT scan and US can detect the mass but differential diagnosis requires a high index of suspicion. 

## Footnotes

**Source of Support:** Nil

**Conflict of Interest:** None declared
